# Organoids: Current Applications and Future Directions

**DOI:** 10.1002/mco2.70965

**Published:** 2026-09-30

**Authors:** Yueqi Leng, Yue Wang, Canhui Cao, Feng Deng, Weisi Lian, Xingtong Chen, Mingmei Lin, Zhonghong Zeng, Dan Mo, Shangqi Li, Yilei He, Yang Yu, Heng Pan, Ping Zhou, Rong Li

**Affiliations:** ^1^ Center For Reproductive Medicine, Department of Obstetrics and Gynecology Peking University Third Hospital Beijing China; ^2^ Key Laboratory of Assisted Reproduction (Peking University) Ministry of Education Beijing China; ^3^ Beijing Key Laboratory of Reproductive Endocrinology and Assisted Reproductive Technology Beijing China; ^4^ National Clinical Research Center for Obstetrics and Gynecology (Peking University Third Hospital) Beijing China; ^5^ Department of Obstetrics and Gynecology, Union Hospital, Tongji Medical College Huazhong University of Science and Technology Wuhan Hubei China; ^6^ China Center of Industrial Culture Collection China National Research Institute of Food and Fermentation Industries Co., Ltd Beijing China; ^7^ National Center for Clinical Laboratories, Institute of Geriatric Medicine, Chinese Academy of Medical Sciences Beijing Hospital/National Center of Gerontology Beijing China; ^8^ Chinese Academy of Medical Sciences and Peking Union Medical College Beijing China; ^9^ Clinical Stem Cell Research Center Peking University Third Hospital Beijing China

**Keywords:** clinical translation, disease modeling, organoids, precision medicine, regenerative medicine

## Abstract

Organoids are three‐dimensional multicellular structures derived from stem cells or primary tissues that recapitulate key structural and functional features of native organs. Advances in stem cell biology, biomaterials, and bioengineering have established organoids as powerful platforms for studying human development and disease mechanisms, drug discovery, precision medicine, and regenerative medicine. However, current organoid models are limited by incomplete maturation, insufficient vascularization, inadequate immune and neural integration, and poor reproducibility, restricting their physiological relevance and clinical translation. Moreover, the rapid expansion of organoid research has created a need for an updated synthesis of the technological advances, biological applications, and remaining challenges. This review provides a comprehensive overview of organoid technology, covering strategies for organoid generation, including cell sources, three‐dimensional culture systems, extracellular matrix support, and microenvironment engineering, followed by representative applications of brain, retinal, kidney, cardiac, lung, liver, intestinal, and endometrial organoids. We further discuss current technical bottlenecks and highlight emerging solutions, including organ‐on‐chip platforms, multiorgan integration, multiomics, clustered regularly interspaced short palindromic repeats(CRISPR)‐based engineering, artificial intelligence, standardized biobanks, and clinical translation. By integrating recent advances across diverse organoid systems, this review provides a framework for developing more physiologically relevant and clinically translatable organoid models.

## Graphical abstract

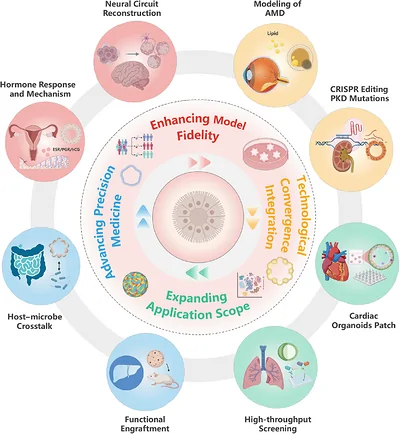

## 1 Introduction

Organoid technology has become an important tool in modern biomedical research. It has transformed the way researchers investigate human development and disease mechanisms, perform drug discovery, and study regenerative medicine [1, 2, 3, 4]. Organoids are three‐dimensional structures generated from stem cells or primary tissues under defined culture conditions [5, 6, 7]. They retain many of the structural and functional features of native organs through intrinsic self‐organization [8, 9].

The evolution of organoid technology has been driven by a series of landmark advances that have progressively transformed it from a conceptual framework into a versatile platform for biomedical research. The concept of cellular self‐organization originated in 1907, when Wilson demonstrated that dissociated sponge cells could spontaneously reaggregate into organized tissues, providing the theoretical foundation for organoid development [10]. In 1987, the introduction of Matrigel established a biologically relevant three‐dimensional culture matrix [11], and in 2006, the development of induced pluripotent stem cell (iPSC) technology further expanded the range of cellular sources available for organoid generation [12, 13, 14]. A defining milestone was achieved in 2009, when Clevers et al. [15] generated long‐term intestinal organoids from single Lgr5^+^ adult stem cells, establishing the first modern organoid culture system and providing a universal strategy for epithelial organoid generation [16]. The following years witnessed the rapid expansion of organoid models: gastric organoids were established in 2010 [17], human colorectal cancer (CRC) organoids and retinal organoids were reported in 2011 [18, 19]; in 2013, vascularized iPSC‐derived liver buds and cerebral organoids further expanded organoid technology from epithelial self‐renewal systems toward organ‐bud engineering and regenerative medicine [20, 21]. By 2015, standardized kidney organoids and functional airway and alveolar organoids had been established [22, 23], greatly extending the application of organoids to developmental biology and disease modeling. The establishment of the first patient‐derived organoid (PDO) biobank before 2016 marked another major step toward precision oncology and personalized medicine [24, 25]. Since 2018, the field has entered an era focused on engineering and translation. Vascularized‐transplanted brain organoids [26], multiorgan organ‐on‐chip systems [27], and artificial intelligence(AI)‐enabled organoid platforms have substantially improved the physiological relevance and experimental scalability of this technology. More recently, reproductive organoids, clinically guided PDO‐based drug sensitivity testing, and bioprinted vascularized composite organoids have further accelerated the translation of organoid technology from laboratory research to regenerative medicine and precision therapy (Figure 1) [28, 29, 30, 31].

**FIGURE 1:**
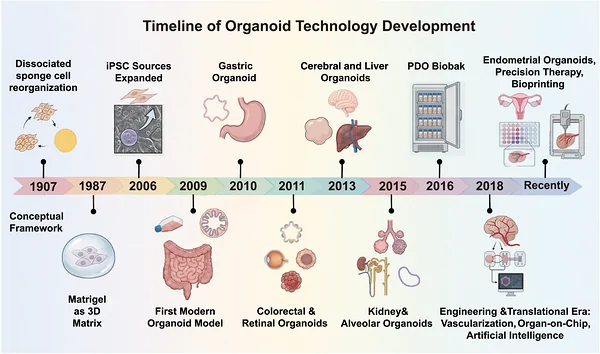
Timeline of organoid technology development. Key evolutionary milestones cover the construction of 3D extracellular matrix culture platforms, the development of induced pluripotent stem cell technology, the first long‐term epithelial organoid protocols, the generation of diverse tissue‐specific organoids including cerebral, liver, gastric, colorectal, retinal, kidney, lung, and endometrial models together with vascularized organ buds, patient‐derived organoid biobanks, organ‐on‐chip systems, multiorgan integration, and AI‐assisted organoid engineering. This chronological roadmap traces the field from foundational cell self‐organization to modern multitissue, clinically oriented organoid engineering.

Although organoid research has advanced rapidly, recent reviews have often been organized around a single organ, disease, or technology, with relatively little emphasis on the common principles that underlie different organoid systems. The aim of this review is to provide a broader perspective by integrating recent advances across multiple organ systems and identifying shared opportunities and challenges in organoid research. Its major strength lies in the comparison of representative organoid models across multiple organ systems to identify common cultivation strategies, universal technical bottlenecks, and emerging engineering solutions, thereby offering a broader framework for the future development and clinical application of organoid technology.

To present these advances in a coherent framework, this review is organized according to the developmental progression of organoid technology. We first summarize the overall strengths and current bottlenecks of organoid systems, followed by the fundamental considerations underlying organoid establishment, including cell source selection, culture media, three‐dimensional culture techniques, support materials, and microenvironment simulation. We then examine representative applications across major organ systems and summarize their biological and translational significance. Building on these applications, we discuss the key challenges that continue to limit organoid fidelity and clinical implementation, including insufficient vascularization, the absence of immune and neural components, heterogeneity, and poor standardization. Finally, we outline future directions focused on improving physiological relevance through multiorgan integration, technological convergence with multiomics, CRISPR, and artificial intelligence (AI), as well as standardized biobanks, clinical translation, and ethical considerations.

## 2 Overview of Organoid Technology: Strengths and Bottlenecks

Organoid technology provides a three‐dimensional, self‐organizing experimental platform that bridges the gap between conventional two‐dimensional culture and in vivo physiology [1, 2, 3, 4, 32]. Through the integration of suitable cell sources, well defined morphogen and growth factor signals, as well as extracellular matrix (ECM) scaffolds, organoids are capable of reconstructing core features including tissue structural organization, cell fate determination, intercellular signaling, and functional characteristics unique to each organ type [33]. Compared with monolayer cultures, organoids better preserve spatial organization, cellular heterogeneity, polarity, and patient‐specific genetic or phenotypic characteristics, making them particularly useful for studying human development, disease initiation, drug response, and regenerative potential across multiple organ systems [34, 35]. These advantages are especially evident in contexts where primary tissues are scarce, animal models incompletely reproduce human biology, or longitudinal sampling is difficult, such as in the context of neurodevelopmental disorders, cancer precision medicine, host–pathogen interactions, inherited diseases, and reproductive medicine [36, 37, 38].​

Despite these strengths, these organoids should not be regarded as complete miniature organs. Most current systems still capture only selected epithelial, parenchymal, or lineage‐restricted compartments, while vascular perfusion, immune surveillance, neural regulation, stromal remodeling, and mechanical forces are often absent or only partially reconstructed [39, 40, 41, 42]. As organoid size or culture duration increases, limited diffusion of oxygen and nutrients can lead to hypoxia, necrosis, and incomplete maturation, thereby affecting functional readouts and translational reliability [43, 44, 45, 46]. Furthermore, numerous existing culture protocols remain dependent on undefined native matrix materials including Matrigel. These substances generate inconsistent outcomes across different production batches and hinder the establishment of standardized systems suitable for clinical applications [47, 48, 49, 50]. Variations derived from distinct tissue donors, variable cell passage counts, differing culture periods, divergent cell differentiation states, and laboratory exclusive culture protocols collectively impair experimental reproducibility and hinder consistent large scale comparative analysis [51]. Accordingly, further advancement of organoid research necessitates fully defined matrix materials, coculture systems featuring vascular structures and functional immune components, organ‐on‐chip devices, unified quality control benchmarks, as well as synergistic combinations with multiomics profiling, CRISPR‐mediated gene manipulation, AI tools, and clinical tissue biobanks with complete clinical annotation records [34, 52].

## 3 The Cultivation of Organoids

Organoid cultivation relies on the selection of appropriate cell sources (embryonic stem cells (ESCs), iPSCs, adult stem cells, etc.), the optimization of culture media supplemented with growth factors and cytokines combined with 3D culture techniques (including natural/synthetic matrix‐based systems and dynamic microfluidic platforms), and the use of natural or synthetic scaffold materials integrated with ECM components to precisely simulate the in vivo microenvironment, all of which are crucial for constructing functional organoid models.

### 3.1 Cell Source and Selection

During organoid construction, the source and quality of the cells are the fundamental prerequisites for ensuring the accuracy of the model [53]. The common cell sources include the following: ESCs, iPSCs, and adult stem cells [54]. ESCs possess totipotency, meaning that they can be induced to differentiate into various types of tissues and organs in vitro [53]. Their advantage lies in their high differentiation potential, but they are also associated with certain ethical and clinical transformation‐related controversies and limitations [55]. iPSCs are obtained by reprogramming somatic cells, avoiding the ethical controversy associated with ESCs, and they possess high differentiation capabilities [32]. Organoids constructed using iPSCs can not only reflect individual specificity but also enable the construction of personalized disease models [56]. Adult stem cells and other primary cells have tissue‐specific origins, such as primary cells from organs such as the liver and the intestine, which can have more similar functions and physiological states as the original organs [54]. Although these cells possess relatively constrained capacity for proliferation and differentiation, they exhibit distinct strengths in disease simulation, drug response testing, and research targeting tissue regenerative mechanisms [57].

### 3.2 Culture Medium and 3D Culture

The selection and preparation of culture media play crucial roles in cell differentiation and organ formation during the process of constructing organoids [58]. Traditional two‐dimensional culture media can no longer meet the demands of complex three‐dimensional structures; thus, three‐dimensional culture techniques have been widely applied [59]. Culture media usually require the addition of growth factors, cytokines, and auxiliary molecules [15]. These components not only provide the nutrients needed for cell growth but also regulate the self‐organization process of cells through signal transduction pathways [33]. In recent years, culture medium optimization methods based on high‐throughput screening have emerged continuously, providing a scientific basis for the construction of functional organoids [60]. Traditional two‐dimensional culture is limited by cells being in planar contact, whereas three‐dimensional culture can be used to establish a three‐dimensional cellular environment through natural or synthetic matrices [61]. Common organoid three‐dimensional culture systems include culture methods based on natural matrices such as collagen and Matrigel, as well as synthetic scaffolds such as polylactic‐co‐glycolic acid [62]. Three‐dimensional cell culture not only facilitates direct interaction between cells but also promotes rich ECM structure and signaling in the in vivo microenvironment [63]. The dynamic culture platform constructed by using microfluidic technology can simulate in vivo fluid circulation and hemodynamic conditions, further enhancing the physiological relevance of organoid models [64]. The dynamic culture system can provide real‐time nutrition, allow waste removal, and provide growth factor gradients, promoting the formation and functional development of complex networks within organoids [65].

### 3.3 Support Material and Microenvironment Simulation

The success of organoid cultivation relies on the precise simulation of the physiological microenvironment within the body [34]. The scaffold material serves as the basis for three‐dimensional cultivation, and its physical, chemical, and biological compatibility directly affect the adhesion, proliferation, and differentiation of cells [35]. The scaffold materials can be classified into two major categories: natural materials and synthetic materials [66]. Natural materials such as collagen, fibrin, and gelatin are widely used because of their biocompatibility and bioactivity [32]; synthetic materials such as polycaprolactone and polylactic acid achieve the desired mechanical properties and stability via changes in structural parameters [67]. Advanced 3D bioprinting and microprocessing technologies can generate scaffolds with complex pore structures and gradient changes according to specific requirements [68]. Such scaffolds enable a more natural spatial distribution of cells during the cultivation process and allow precise control of local growth factor concentrations via adjustment of parameters such as pore density and surface morphology [68]. ECM is important for maintaining organ functions, and its components are complex and diverse. In modern organoid cultivation, some ECM components, such as glycosaminoglycans and elastin, are often incorporated into scaffold materials to simulate the interaction between cells and the matrix in the body, thereby promoting the complete expression of cell functions [69].

Organoid cultivation relies on the selection of appropriate cell sources (ESCs, iPSCs, adult stem cells, etc.), the optimization of culture media supplemented with growth factors and cytokines combined with 3D culture techniques (including natural/synthetic matrix‐based systems and dynamic microfluidic platforms), and the use of natural or synthetic scaffold materials integrated with ECM components to precisely simulate the in vivo microenvironment, all of which are important for constructing functional organoid models.

## 4 Current Applications of Organoids in Organ Systems

The use of organoid technology has led to remarkable breakthroughs in biomedical research, with extensive and in‐depth applications across key organs of multiple physiological systems, including the brain, retina, kidney, heart, lung, liver, intestine, and endometrium, providing powerful tools for dissecting tissue physiology, modeling diseases, and advancing translational medicine.

### 4.1 Brain Organoids

The brain is a highly complex organ composed of neurons, glial cells (astrocytes, oligodendrocytes, and microglia), and intricate neural networks that govern cognitive functions, sensory perception, motor control, and emotional regulation through electrochemical signaling and region‐specific functional specialization.

Brain organoids have emerged as versatile and powerful tools that have transformed multiple facets of neuroscience research and translational medicine, with applications spanning disease modeling, neural circuit reconstruction, drug discovery, regenerative therapy, and personalized medicine [70]. In disease modeling, brain organoids faithfully recapitulate key features of both genetic and acquired neurological disorders; for instance, cerebral organoids derived from patient‐iPSCs have enabled mechanistic investigations of microcephaly, Down syndrome, and Timothy syndrome, whereas infection models have elucidated the neurotropism and pathogenesis mechanisms of viruses such as Zika virus, severe acute respiratory syndrome coronavirus 2 (SARS‐CoV‐2), and herpes simplex virus, revealing virus‐induced neuronal death, synaptic loss, and neuroinflammatory responses [71].

Notably, brain organoids and their assembloids, generated by fusing region‐specific organoids (e.g., cortical, thalamic, and striatal), have revolutionized the study of neural circuitry, recapitulating interneuron migration, axonal projection, and functional synaptic connectivity between brain regions, thus providing unprecedented insights into corticothalamic, nigrostriatal, and corticospinal circuit development and dysfunction in conditions such as Parkinson's disease and Huntington's disease [72]. In drug discovery, these 3D models offer a more physiologically relevant alternative to traditional 2D cultures and animal models, facilitating high‐throughput screening for neuroprotective agents, antiepileptic drugs, and therapies targeting neurodegenerative diseases (e.g., Alzheimer's disease‐related amyloid‐aggregation and tau phosphorylation) while also enabling the assessment of neurotoxicity to reduce translational failure [73]. In addition, brain organoids hold promise in regenerative medicine: preclinical studies have demonstrated successful transplantation of brain organoids into rodent brains, where they integrate with host neural circuits, undergo vascularization, and promote functional recovery in models of brain injury, laying the groundwork for potential therapeutic applications in stroke, traumatic brain injury, and retinal degeneration [74]. Additionally, patient‐derived brain organoids preserve the genomic and phenotypic heterogeneity of individual tumors (e.g., glioblastoma), enabling personalized drug sensitivity testing and the development of tailored treatment strategies, thereby bridging the gap between bench research and clinical care [75].

Collectively, these applications underscore the unique ability of brain organoids to elucidate human‐specific neural development and disease mechanisms, accelerate drug development, and advance precision neuroscience.

### 4.2 Retinal Organoids

The retina, a specialized neural tissue lining the posterior eye, is critical for vision, as it converts light into electrical signals via a complex laminar structure comprising photoreceptors, bipolar cells, retinal ganglion cells (RGCs), Müller glia, and retinal pigment epithelium (RPE), all of which coordinate to transmit visual information to the brain [76].

Retinal organoids have emerged as transformative tools in ophthalmic research and translational medicine, bridging the gap between traditional 2D cell cultures and in vivo models by recapitulating this intricate architecture and cellular diversity [77]. Their applications span disease modeling, drug discovery, regenerative therapy, and precision medicine, addressing unmet needs in studying retinal development and vision disorders [78]. In disease modeling, patient‐derived retinal organoids faithfully replicate key pathological features of both genetic and acquired retinal diseases; for instance, organoids harboring cone‑rod homeobox(CRX) mutations recapitulate Leber's congenital amaurosis, while those with eyes shut homolog gene mutations mimic retinitis pigmentosa, enabling mechanistic investigations into photoreceptor degeneration and synaptic loss [79]. Additionally, retinal organoids have been instrumental in modeling age‐related macular degeneration (AMD) and Stargardt disease, recapitulating hallmark phenotypes such as RPE dysfunction and lipid accumulation [80].

In drug discovery and toxicity assessment, these 3D models offer enhanced physiological relevance compared with conventional systems, facilitating high‐throughput screening of neuroprotective agents, gene therapy vectors, and small molecules that target retinal degeneration; for example, compounds that mitigate A aggregation and tau phosphorylation in AMD models have been identified [81]. Notably, advancements in multimodal integration (combining single‐cell transcriptomics, chromatin accessibility, and multiplexed imaging) have enabled the reconstruction of gene regulatory networks underlying retinal development, highlighting key transcription factors such as orthodenticle homeobox 2 and CRX that govern photoreceptor fate, thereby guiding targeted drug development [82]. Preclinical studies have further validated their potential for restoring vision: transplanted organoids integrate with host retinal circuits, mature into functional photoreceptors, and preserve retinal structure in animal models of retinal degeneration [83].

Moreover, retinal organoids facilitate precision medicine through the establishment of patient‐specific biobanks, enabling personalized drug sensitivity testing and the development of tailored gene therapy strategies, for example, correcting cystic fibrosis transmembrane conductance regulator(CFTR) mutations in cystic fibrosis‐related retinal dysfunction via CRISPR editing [84]. When combined with organ‐on‐a‐chip technology, these methods recapitulate vascular perfusion and intercellular crosstalk, further enhancing their translational utility [85].

Taken together, the results of this study reveal that retinal organoids continue to revolutionize our understanding of retinal biology, accelerate the development of novel therapeutics, and pave the way for curative treatments for previously untreatable vision disorders.

### 4.3 Kidney Organoids

The kidney develops from the reciprocal interactions between the metanephric mesenchyme and ureteric bud, which undergo sequential proliferation, differentiation, and morphogenesis to form functional nephrons, alongside supportive stromal and vascular networks [86]. Kidney organoids have emerged as powerful tools for transforming renal research and translational medicine, recapitulating key developmental processes and cellular heterogeneity to address unmet needs in disease modeling, drug discovery, regenerative therapy, and developmental biology [87].

In disease modeling, kidney organoids excel at recapitulating genetic and acquired renal disorders: CRISPR‐edited organoids with polycystin‑1(PKD1) or polycystin‑2(PKD2) mutations faithfully replicate cystogenesis in autosomal dominant polycystic kidney disease (ADPKD), enabling mechanistic studies of cyst initiation and expansion, while PDOs model congenital anomalies of the kidney and urinary tract (CAKUT) and diabetic nephropathy by preserving patient‐specific genomic and phenotypic features [88]. Notably, scalable organoid platforms have facilitated high‐throughput drug screening, identifying cyst‐inhibiting compounds such as QNZ(EVP‑4593), histone deacetylase inhibitors, and the B‑cell‑specific moloney murine leukemia virus integration site 1 inhibitor PTC‐209, which suppress cyst growth in ADPKD models without compromising organoid viability [89]. In regenerative medicine, advancements in ECM engineering, including decellularized kidney ECM hydrogels derived from the porcine or human renal cortex, have enhanced organoid vascularization and maturation, with transplanted organoids integrating with host vasculature and demonstrating glomerular filtration function in preclinical models [90].

Additionally, long‐term expandable nephron progenitor cell (NPC) lines derived from pluripotent stem cells cells have enabled in‐depth exploration of renal developmental plasticity, such as podocyte‐to‐NPC reprogramming, and the development of genome‐wide CRISPR screens that reveal key genes that regulate nephrogenesis and disease pathogenesis (e.g., mTOR signaling, epigenetic regulators lysine methyltransferase 2A and lysine acetyltransferase 6A) [91]. When combined with microfluidics or 3D bioprinting, kidney organoids further recapitulate physiological microenvironments, including fluid flow and interorgan crosstalk, which has enhanced their utility for preclinical toxicity testing and personalized medicine [92].

In summary, these applications underscore the unique capacity of kidney organoids to bridge the gap between in vitro research and in vivo physiology, accelerating our understanding of renal biology, enabling targeted drug development, and paving the way for the development of regenerative therapies for end‐stage renal disease and other refractory renal conditions.

### 4.4 Cardiac Organoids

The heart, as a complex muscular organ composed of cardiomyocytes, endothelial cells, and cardiac fibroblasts, functions as the central pump of the circulatory system, maintaining systemic blood flow and oxygen transport through rhythmic contraction and electrophysiological coordination [93]. Cardiac organoids have emerged as transformative tools in cardiovascular research and translational medicine, recapitulating key structural, functional, and pathological features of the human heart to address unmet needs in disease modeling, drug development, regenerative therapy, and toxicology assessment—findings strongly supported by recent studies [94].

In disease modeling, cardiac organoids excel at replicating myocardial infarction (MI), and human cardiac organoids have been used to simulate the hypoxic‐acidic microenvironment post‐MI (pH ≈5.8), verifying that coadministration of eggshell microparticles and exosomes can regulate local pH, reduce cardiomyocyte mortality, and preserve proliferative capacity, laying the foundation for the development of self‐driven microneedle patches that deliver vascular endothelial growth factor (VEGF) and exosomes to reconstruct vascular networks and rescue infarcted myocardium in preclinical models [95]. Additionally, they have been employed to model congenital heart defects associated with gestational hyperglycemia, arrhythmias induced by drugs such as ondansetron, and cardiac fibrosis, with multichamber cardiac organoids uncovering chamber‐specific defects caused by genetic mutations (e.g., ISL LIM homeobox 1, T‑box transcription factor 5) [96]. In toxicological assessment, cardiac organoid‐on‐a‐chip platforms have been employed to dynamically evaluate polystyrene nanoparticle (PS‐NP)‐induced cardiotoxicity, revealing that short‐term exposure triggers oxidative stress, disruption of calcium homeostasis, and mitochondrial dysfunction, while long‐term exposure leads to cardiac fibrosis, with low‐dose PS‐NPs even exacerbating MI symptoms in pathological models [97].

For drug discovery and regenerative therapy, engineered cardiac organoid patches have demonstrated regenerative potential in repairing ventricular function posttransplantation, and microfluidic integration has enhanced their utility for high‐throughput screening of cardioprotective agents such as metformin to reverse mitoxantrone‐induced arrhythmias [98]. Furthermore, multiorganoid systems such as cardiorenal and cardiopulmonary organoids have been developed to simulate interorgan crosstalk, advancing the understanding of systemic disease mechanisms such as cardiorenal syndrome [99].

Overall, these applications, rooted in rigorous experimental evidence from recent studies, underscore the unique capacity of cardiac organoids to bridge in vitro research and in vivo physiology, accelerating the acquisition of mechanistic insights into cardiovascular diseases and paving the way for the development of personalized and curative therapies.

### 4.5 Lung Organoids

The lung is a respiratory organ comprising proximal airways and distal alveoli and enables gas exchange via specialized epithelial lineages (e.g., alveolar type 1, alveolar type 2, and basal cells) while coordinating with mesenchymal and immune components to maintain tissue homeostasis and respond to injury [100]. Lung organoids have emerged as transformative tools in respiratory research and translational medicine, recapitulating key structural and functional features of the human lung to address unmet needs in disease modeling, drug discovery, regenerative therapy, and developmental biology—findings strongly supported by recent studies [101]. In developmental biology, organoids derived from fetal lung tip progenitors (CD44^+^CD36^+^ cells) have helped elucidate mechanisms of alveolar fate specification, revealing that Wnt signaling from fibroblasts and spatial patterning by notum, palmitoleoyl‑protein carboxylesterase‐secreting myofibroblasts regulate alveolar type 2 (AT2) cell differentiation, with the transcription factor NK2 homeobox 1(NKX2) [102], serving as a central driver of alveolar maturation and surfactant production [85].

For disease modeling, they faithfully replicate genetic and acquired respiratory disorders: PSC‐derived or primary AT2 cell organoids have been instrumental in the study of SARS‐CoV‐2 infection, demonstrating viral tropism for AT2 cells and revealing mechanisms of epithelial damage and inflammatory responses [103]. Organoids harboring NKX2 mutations recapitulate interstitial lung disease phenotypes, whereas models of fibrotic lung disease mimic epithelial‒mesenchymal crosstalk and ECM accumulation [87].

In drug discovery, lung organoids offer a physiologically relevant platform for high‐throughput screening: they have been used to identify anti‐inflammatory compounds that target interleukin‑1 and tumor necrosis factor signaling in acute lung injury, as well as inhibitors to modulate Wnt activity in fibrotic models [104]. In regenerative medicine, AT2 cell organoids (alveolospheres) have shown promise in preclinical studies, with transplantation into injured lung models promoting alveolar repair by replenishing functional AT2 cells and supporting surfactant secretion [105]. Additionally, integration with advanced technologies has enhanced their utility: microfluidic‐based organ‐on‐a‐chip systems enable the simulation of lung biomechanics and vascular perfusion, while CRISPR–Cas9 editing in organoids enables precise dissection of disease‐causing mutations [106].

In essence, these applications, rooted in rigorous experimental evidence from recent studies, underscore the unique capacity of lung organoids to bridge in vitro research and in vivo physiology, advancing our understanding of respiratory biology and paving the way for the development of personalized therapies for lung diseases, ranging from infections to fibrosis and genetic disorders.

### 4.6 Liver Organoids

As a central metabolic and detoxifying organ in the human body, the liver performs diverse essential functions, including nutrient metabolism, bile secretion, toxin clearance, and plasma protein synthesis, through the coordinated interplay of hepatocytes, hepatic stellate cells, Kupffer cells, and liver sinusoidal endothelial cells [107]. Liver organoids have emerged as transformative tools in hepatological research and translational medicine, recapitulating key structural and functional features of the human liver to address unmet needs in disease modeling, drug discovery, precision medicine, and regenerative therapy, which is strongly supported by recent studies [108].

In disease modeling, liver organoids excel at replicating metabolic and genetic liver disorders: PSC‐derived or primary hepatocyte organoids have been instrumental in modeling metabolic dysfunction‐associated steatohepatitis (MASH), recapitulating lipid accumulation, inflammatory cytokine release, and hepatic stellate cell activation upon exposure to free fatty acids, whereas CRISPR‐edited organoids harboring patatin‑like phospholipase domain‑containing 3 or glucokinase regulator mutations have elucidated genetic susceptibility to steatosis and mitochondrial dysfunction [109]. They also serve as robust platforms for studying drug‐induced liver injury: liver organoid‐on‐a‐chip systems have successfully predicted synergistic hepatotoxicity of tenofovir–inarigivir combinations and recapitulated acetaminophen‐induced necrosis and fialuridine‐induced steatosis, with enhanced albumin production and cytochrome P450 expression compared with those in 2D cultures [110].

For drug discovery, high‐throughput screening using liver organoids has identified novel targets for steatosis (e.g., fatty acid desaturase 2) via CRISPR loss‐of‐function screens and validated potential therapeutics such as transforming growth factor(TGF) inhibitors for fibrosis [111]. In regenerative medicine, vascularized liver organ buds derived from iPSCs have demonstrated functional engraftment and liver function rescue in animal models of acute liver failure, whereas cholangiocyte organoids have restored biliary function after transplantation in human patients [20]. Additionally, patient‐derived liver organoids preserve donor‐specific genetic and epigenetic profiles, enabling personalized drug sensitivity testing for hepatocellular carcinoma and MASH and facilitating the study of interorgan metabolic crosstalk when integrated with pancreatic islet or adipose tissue organoids in microfluidic systems [112].

In summary, these applications, rooted in rigorous experimental evidence from recent studies, underscore the unique capacity of liver organoids to bridge in vitro research and in vivo physiology, advancing our understanding of liver biology and paving the way for the development of personalized therapies for liver diseases, ranging from metabolic disorders to cancer and fibrosis.

### 4.7 Intestinal Organoids

The intestine is a complex tubular organ responsible for nutrient absorption, immune defense, and host‒microbe crosstalk and comprises diverse epithelial cell types (enterocytes, goblet cells, Paneth cells, and enteroendocrine cells) and a dynamic mucosal microenvironment—features that traditional two‐dimensional cultures fail to recapitulate owing to their lack of structural complexity, cellular heterogeneity, and physiological relevance [113]. Intestinal organoids (including patient‐derived enteroids) have emerged as transformative tools in gastrointestinal research and translational medicine, faithfully replicating intestinal crypt‐villus architecture, genetic signatures, and functional responses to bridge the gap between 2D cultures and animal models—findings strongly supported by recent studies [114].

In infectious disease modeling, intestinal organoids have become gold‐standard platforms for studying enteric pathogens: they enable the replication of previously uncultivatable human noroviruses and sapoviruses, revealing strain‐specific entry mechanisms and host innate immune responses (such as type I/III interferon‐mediated inhibition) that were inaccessible in 2D systems [115]. In terms of bacterial pathogens, details of the pathogenic mechanisms of enterohemorrhagic Escherichia coli O157:H7, including barrier disruption, actin rearrangement, and neutrophil recruitment, have been elucidated in intestinal organoids, and biotin and L‐fucose have been identified as potential therapeutic agents to reduce bacterial attachment [116]. In host‒microbe interaction studies, specialized coculture systems (e.g., mesofluidic platforms with oxygen gradients) have enabled the long‐term cultivation of anaerobic commensals such as Faecalibacterium prausnitzii and Bifidobacterium adolescentis, revealing their anti‐inflammatory effects via butyrate production and their role in intestinal epithelial maturation [117]. Childs et al. leveraged the human intestinal stem cell niche factor epiregulin (EREG) to enhance PSC‐derived human intestinal organoid (HIO) differentiation. EREG‐grown HIOs pattern the epithelium, mesenchyme, smooth muscle, neurons, and endothelium in a single differentiation event. After transplantation, HIOs mature further, and functional studies have shown peristaltic‐like functions and functional vasculature [118].

For disease modeling and precision medicine, PDOs recapitulate the genetic and phenotypic heterogeneity of colorectal cancer (CRC), preserving tumor histology and mutation landscapes to guide personalized drug sensitivity testing— for example, identifying responses to chemotherapeutics and targeted therapies such as hydroxybutyrate [119]. They also model inflammatory bowel disease (IBD) and environmental enteric dysfunction, with autologous organoid–immune cell cocultures shedding light on immune‒bacterial crosstalk in Crohn's disease [120]. In drug discovery and vaccine development, intestinal organoids facilitate high‐throughput screening of antivirals and evaluation of vaccine candidates (e.g., targeting enterotoxigenic Escherichia coli colonization factor colonization factor antigen I), while apical‐out organoid cultures and microfluidic integration overcome the technical limitations of apical access and static culture, which enhances their translational utility [121].

Taken together, these applications underscore the unique ability of intestinal organoids to help elucidate intestinal biology, dissect host–pathogen interactions, and accelerate the development of personalized therapies for gastrointestinal diseases, ranging from infections to cancer and inflammation.

### 4.8 Endometrial Organoids

As the uterine inner lining, the endometrium acts as an indispensable tissue supporting mammalian reproduction, serving as the sole location for embryo implantation, placentation, and fetal development before delivery. Structurally divided into a basalis layer adjacent to the myometrium and a lumen facing functional layer, human endometrium contains glandular epithelial and stromal components that undergo periodic proliferation, differentiation, and shedding driven by cyclic estrogen and progesterone fluctuations across menstrual stages (Figure 2) [122, 123]. This reproductive interface is difficult to capture using conventional 2D cultures, transformed cell lines, or animal models because human endometrial function depends on menstrual cycle‐associated epithelial–stromal remodeling, sex‐steroid responsiveness, immune modulation, vascular adaptation, and implantation‐specific epithelial polarity [124, 125, 126]. Thus, endometrial organoids broaden the conceptual scope of organoid research by extending it from classic developmental, metabolic, and tumor models to a dynamic reproductive tissue with temporally regulated function [127].

**FIGURE 2:**
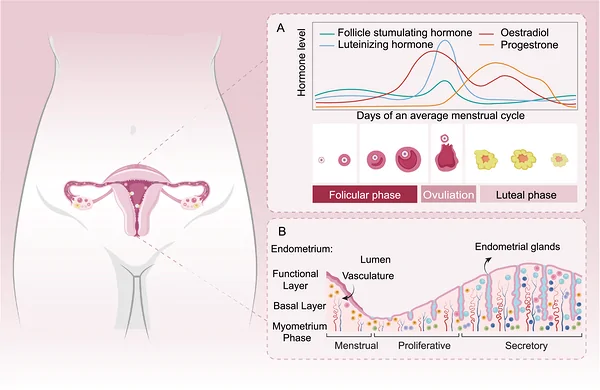
Physiological changes and major hormonal regulation during the menstrual cycle. (A) Cyclic fluctuations of core reproductive hormones, including follicle‑stimulating hormone, luteinizing hormone, estradiol, and progesterone, occur across a complete menstrual cycle. (B) The endometrium undergoes phase‐dependent structural remodeling during menstrual, proliferative, and secretory phases. Hormonal regulation drives periodic morphological changes of the functional layer, basal layer, blood vessels, and endometrial glands.

Endometrial organoid systems were first established from mouse and human endometrial tissue and subsequently shown to maintain glandular epithelial architecture, long‐term expandability, genomic stability, and responsiveness to estrogen and progesterone [128, 129]. Disease‐derived endometrial organoids have further demonstrated that patient‐specific pathological features and clinical heterogeneity can be retained during in vitro expansion, making these models suitable for mechanistic studies and drug screening [130]. Recent methodological advances, including in the development of endometrial assembloids, embryo–endometrial interface models, and vascularized endometrium‐on‐chip platforms, have expanded the field beyond epithelial‐only culture toward more complex multicellular systems [131]. These approaches are particularly important because implantation, decidualization, vascular remodeling, and endometrial repair cannot be fully reproduced without stromal, endothelial, and ECM components [132, 133] (Figure 3).

**FIGURE 3:**
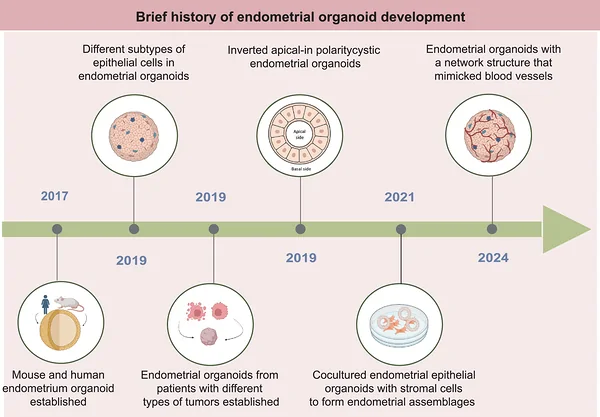
Brief history of endometrial organoid development. Major advances in endometrial organoids research spanning 2017 to 2024 include the initial establishment of mouse and human endometrial organoids, identification of diverse epithelial cell subtypes, generation of patient‐specific organoids derived from tumor samples, establishment of cystic organoids with inverted apical‐in polarity, stromal and epithelial coculture to generate endometrial assembloids, and construction of organoids bearing biomimetic vascular like network structures.

Single‐cell and functional studies have further refined our understanding of endometrial biology and disease‐related epithelial remodeling [134, 135]. These studies have shown that endometrial function is shaped by coordinated signaling among epithelial cells, stromal fibroblasts, immune cells, vascular cells, metabolic programs, and regenerative responses [136, 137, 138, 139, 140]. At the same time, endometrial disease modeling remains challenging because conventional models often fail to preserve hormone responsiveness, cellular heterogeneity, immune–stromal crosstalk, and the dynamic architecture of the native tissue [141, 142]. Therefore, endometrial organoids provide a useful intermediate platform between simplified in vitro culture and complex in vivo physiology [143].

In research on endometriosis, organoid models are especially valuable because the disease involves hormone dependence, chronic inflammation, epithelial–stromal remodeling, immune dysregulation, angiogenesis, fibrosis, and altered endometrial receptivity [144, 145, 146, 147, 148, 149]. Organoids derived from eutopic or ectopic lesions can preserve epithelial disease features, hormone receptor expression, invasion‐like behavior, and disease‐associated molecular signatures, thereby enabling mechanistic investigations of lesion biology [150, 151, 152, 153, 154]. In particular, endometriotic organoid studies have been performed to examine homeobox (HOX) gene methylation, Notch signaling, and epithelial–stromal interactions, providing insight into epigenetic dysregulation, abnormal epithelial proliferation, and altered hormone responsiveness [155, 156, 157, 158].

Endometrial hyperplasia and endometrial cancer represent other major application areas for disease‐derived endometrial organoids [159, 160, 161, 162, 163, 164, 165, 166, 167, 168]. In premalignant and malignant conditions, PDOs can preserve histological features, molecular heterogeneity, mutation profiles, and therapy‐related phenotypes, allowing them to serve as experimentally tractable models for tumor biology and individualized drug testing [168, 169, 170, 171, 172, 173, 174, 175, 176, 177]. Early and recent endometrial cancer organoid studies have shown that patient‐derived cultures can be used for drug sensitivity testing and may reflect clinically relevant treatment responses [177, 178, 179, 180, 181, 182, 183, 184]. The phosphatidylinositol 3‑kinase (PI3K)/threonine kinase (AKT)/phosphatase and tensin homolog (PTEN) axis is particularly relevant in this context, as dysregulation of this pathway is closely associated with endometrial tumorigenesis and provides a rational target for organoid‐based therapeutic assessment [185, 186, 187].

In addition to endometriosis and cancer, endometrial organoid models are being increasingly applied to other benign gynecological disorders [188, 189, 190, 191, 192, 193, 194, 195, 196, 197, 198, 199]. Adenomyosis and endometriosis share several biological features, including hormone dependence, inflammatory remodeling, epithelial–stromal interactions, and impaired reproductive outcomes, making organoid‐based systems potentially useful for dissecting their overlapping and distinct pathogenic mechanisms [200, 201, 202, 203, 204, 205, 206, 207, 208, 209, 210]. Preclinical models and emerging adenomyosis organoids provide additional opportunities to study epithelial‒mesenchymal transition, fibrotic remodeling, and infertility‐associated endometrial dysfunction under more controlled culture conditions [211, 212, 213, 214, 215, 216, 217, 218].

With respect to polycystic ovary syndrome‐related endometrial dysfunction, organoids offer a platform to separate systemic endocrine effects from intrinsic endometrial abnormalities [219, 220, 221, 222, 223, 224, 225, 226, 227, 228, 229, 230]. Scaffold‐free or patient‐derived endometrial organoids exposed to androgen excess or derived from patients with PCOS can exhibit altered epithelial programs, impaired expression of receptivity‐related markers, and metabolic or transcriptomic dysregulation [230, 231]. These findings support the use of endometrial organoids for identifying disease‐associated biomarkers and testing targeted interventions in hormone‐ and metabolism‐related reproductive disorders [232, 233].

In regenerative contexts, a thin endometrium and intrauterine adhesion represent clinically important but difficult‐to‐model conditions [234, 235, 236, 237, 238, 239, 240, 241, 242, 243, 244]. Organoid‐based studies of thin endometria have begun to reveal epithelial cytoskeletal remodeling, intercellular junction disruption, metabolic stress responses, and impaired repair capacity under injury‐like conditions [245]. In parallel, organoid transplantation has been explored as a preclinical strategy to promote endometrial repair and improve reproductive prognosis in mouse models [246]. These studies suggest that endometrial organoids may function not only as disease models but also as regenerative units or screening platforms for evaluating tissue repair strategies [247, 248].

Therefore, within a comprehensive review of organoids, endometrial organoids should be viewed as an emerging but strategically important model rather than a fully mature mainstream platform. Their main scientific contribution lies in demonstrating how organoid technology can be adapted to hormone‐responsive, cyclically remodeling, implantation‐competent, and injury‐prone reproductive tissues. The studies summarized in Table 1 show that endometrial organoids have begun to bridge basic disease modeling and translational applications across several mechanistic directions [124, 130, 131, 132, 133]. Mitochondrial rescue, epithelial–stromal interactions, vascular endothelial growth factor (VEGF)‐mediated angiogenesis, PI3K/AKT/PTEN signaling, estrogen receptor (ER)/progesterone receptor (PR)‐mediated hormone cycling, metabolic or transcriptomic dysregulation, and fibrosis‐related regeneration together illustrate how organoid systems can be used to investigate endometrial repair, receptivity, disease progression, and therapeutic response [155, 156, 157, 158, 181, 182, 183, 184, 185, 186, 187]. Although these models remain less mature than intestinal, cerebral, hepatic, or renal organoid platforms are, their value lies in adapting organoid technology to hormone‐responsive, cyclically remodeling, implantation‐competent, and injury‐prone reproductive tissue [36, 37, 39, 40, 41, 42, 43, 44, 45]. Future progress will depend on the integration of CRISPR‐based perturbation, vascularization strategies, long‐term culture systems, disease‐specific biobanks, synthetic matrices, and organ‐on‐chip or embryo–endometrial interface platforms [46, 47, 48, 49, 50]. In this context, endometrial organoids provide a focused example of how organoid platforms can advance reproductive medicine while also highlighting common challenges shared across organ systems, including incomplete multicellular complexity, limited vascular and immune integration, insufficient standardization, and the need for stronger translational validation [51, 52] (Figure 4).

**TABLE 1:** Representative preclinical and translational studies of endometrial organoids highlighting major signaling pathways and mechanisms.

Strategy/pathway	Experimental model	Intervention	Mechanisms of action	Main outcome/therapeutic relevance	References
Mitochondrial rescue (oxidative phosphorylation/mitochondrial dysfunction)	Mouse uterine injury model (preclinical)	Transfer of mitochondria derived from endometrial organoids into injured uterine tissue	Restores energy metabolism; reduces apoptosis; enhances epithelial and stromal repair	Improved structural repair and partial restoration of fertility metrics	[124]
Epithelial–stromal interaction/angiogenic signaling (VEGF pathway)	Mouse uterine injury model (preclinical)	Transplantation of autologous/homologous uterine epithelial organoids (EEOs) into the damaged uterus	Stromal–epithelial paracrine repair; activation of VEGF‐mediated angiogenesis and epithelial proliferation	Engrafted organoids integrated into host tissue and supported vascularized endometrial regeneration	[42, 133, 248]
PI3K/AKT/PTEN/oncogenic mutation pathway	Orthotopic xenograft of patient‐derived endometrial cancer organoids in mice	Implantation of tumor organoids + drug sensitivity assays	Targeted inhibition of PI3K signaling leads to reduced proliferation, increased apoptosis	Drug responses in organoids correlated with in vivo tumor response, potential personalized therapy predictor	[130, 168, 170, 174, 175, 176, 177]
Hormone receptor signaling (ER/PR)/endometrial cyclicity	Long‐term human endometrial organoids with hormone cycling (in vitro/ex vivo)	Estrogen + progesterone cycling exposure over prolonged culture	Maintains hormone responsiveness; cyclic proliferation/differentiation mimicry	Validates physiological relevance; provides platform for testing endocrine therapies	[43, 125, 127, 129]
Metabolic and transcriptomic dysregulation in benign disease	Patient‐derived organoids (in vitro)	Culture of organoids from PCOS patients + transcriptomic profiling	Identifies altered gene expression; functional defects in receptivity markers	Supports using organoids for identifying biomarkers and testing targeted interventions in PCOS	[44, 166, 219, 220, 221, 222]
Fibrosis, inflammation, and regenerative pathways	Human observational/biobank study	Establish a 3D endometrial organoid library derived from IUA/endometrial injury patients	Development of organoid biobanks from diseased endometrium for mechanism and drug‐screening research	Supports translational readiness and resource standardization; not yet interventional	[42, 52, 140, 143, 234, 235, 236]

Abbreviations: AKT, protein kinase; ECM, extracellular matrix; EEOs, endometrial epithelial organoids; ER, estrogen receptor; IUA, intrauterine adhesion; PCOS, polycystic ovary syndrome; PI3K, phosphoinositide 3‐kinase; PR, progesterone receptor; PTEN, phosphatase and tensin homolog; VEGF, vascular endothelial growth factor.

**FIGURE 4:**
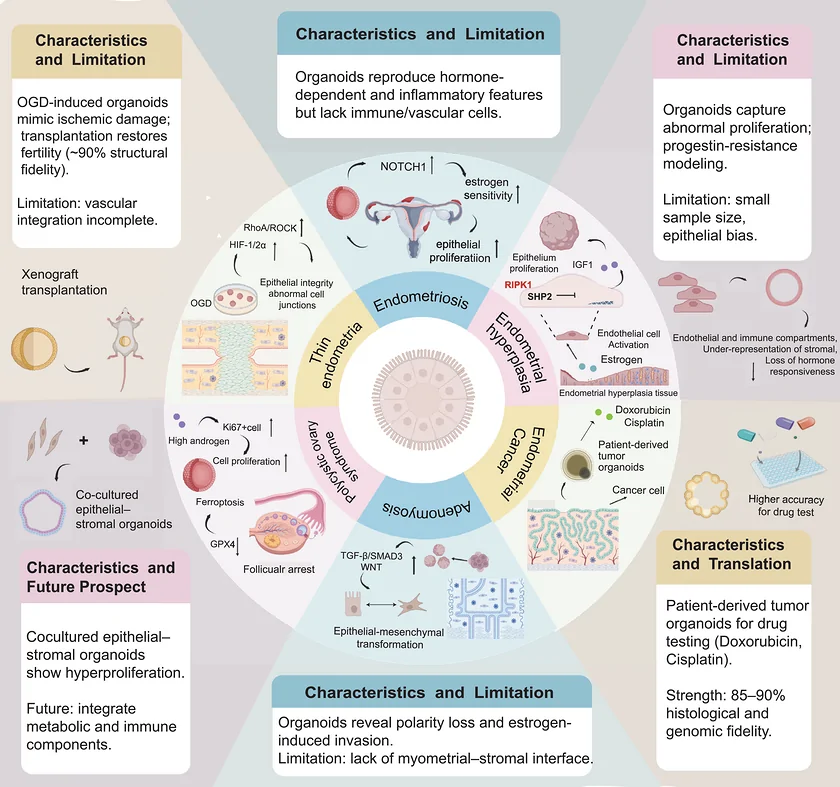
Application and mechanism of endometrial organoids in reproductive diseases. Endometriosis organoids model progesterone resistance and lesion invasion via abnormal epigenetic and inflammatory signaling. Endometrial hyperplasia organoids simulate estrogen‐dependent premalignant proliferation. Endometrial cancer organoids preserve tumor mutation features to study PI3K/AKT pathway targeted therapy. Adenomyosis organoids recapitulate epithelial–mesenchymal transition and uterine fibrosis. Polycystic ovary syndrome‐derived organoids reveal androgen‐mediated endometrial receptivity defects. Thin endometrium organoids mimic injury‐induced metabolic disorder for regenerative mechanism research.

Taken together, the organoid models demonstrate the broad applicability of this technology across multiple organ systems while also revealing organ‐specific strengths and limitations (Table 2). Brain and retinal organoids are particularly valuable for modeling human‐specific neural development, inherited degeneration, neurotoxicity, and gene therapy responses, although maturation, vascularization, synaptic integration, and ethical issues remain important concerns. Kidney, cardiac, lung, intestine and liver organoids provide physiologically relevant platforms for studying organ development, genetic disease, tissue injury, fibrosis, toxicity, and regenerative repair, but their translational potential is still limited by incomplete vascular perfusion, insufficient adult‐like maturation, and limited incorporation of immune or stromal components. Intestinal organoids represent among the most well‐established systems, with strong applications in host–pathogen interactions, microbiota research, inflammatory disease, cancer modeling, CFTR functional testing, and drug or vaccine screening [249]. In contrast, endometrial organoids are still emerging but offer a distinctive reproductive‐medicine platform for studying hormone‐responsive epithelial remodeling, implantation, endometriosis, endometrial cancer, and regenerative repair in thin endometria or intrauterine adhesion. Overall, these eight organoid models collectively illustrate how organoid technology can bridge conventional in vitro systems and in vivo physiology while underscoring the shared need for improved maturation, multicellular complexity, reproducibility, and clinical standardization.

**TABLE 2:** Major features, representative applications, translational value, and limitations of organoid models across organ systems.

Organ system	Key model features	Representative applications	Translational value/main bottleneck	References
Brain	Region‐specific neural differentiation; assembloids can model interregional connectivity.	Neurodevelopmental disorders, viral infection, neurodegeneration, tumor modeling, neurotoxicity screening.	Human‐specific neural modeling; limited vascularization, maturation, microglia, and ethical boundaries.	[70, 71, 72, 73, 74, 75]
Retina	Layered retinal cell types including photoreceptors, RGCs, Müller glia, and RPE‐like components.	Inherited retinal degeneration, AMD/Stargardt models, gene therapy testing, transplantation studies.	Supports precision ophthalmology; challenges include maturation, synaptic integration, and vascular/immune modeling.	[76, 77, 78, 79, 80, 81, 82, 83, 84, 85]
Kidney	Nephron‐like structures with podocyte, tubular, stromal, and emerging vascular components.	ADPKD/CAKUT modeling, nephrotoxicity, CRISPR screens, regenerative studies.	Useful for renal disease mechanisms and drug screening; limited vascular maturation and filtration fidelity.	[86, 87, 88, 89, 90, 91, 92]
Cardiac	Cardiomyocytes with endothelial and fibroblast components; beating and electrophysiological phenotypes.	Myocardial infarction, arrhythmia, cardiotoxicity, fibrosis, regenerative patches.	Strong disease/toxicity platform; requires improved chamber patterning, perfusion, and adult‐like maturation.	[93, 94, 95, 96, 97, 98, 99]
Lung	Airway/alveolar epithelial lineages with optional mesenchymal or chip‐based mechanical cues.	SARS‐CoV‐2 infection, fibrosis, genetic lung disease, acute injury, drug screening.	Models respiratory injury and repair; immune, vascular, and mechanical complexity remain incomplete.	[100, 101, 102, 103, 104, 105, 106]
Liver	Hepatocyte/cholangiocyte‐like lineages with metabolic and detoxification functions.	MASH, drug‐induced liver injury, fibrosis, hepatocellular carcinoma, transplantation studies.	Valuable for metabolism and toxicity; maturation, zonation, Kupffer/LSEC integration remain bottlenecks.	[20, 107, 108, 109, 110, 111, 112]
Intestine	Crypt‐villus‐like epithelial architecture; apical‐out and coculture variants improve access.	Host–pathogen interaction, microbiota studies, IBD, CRC, CFTR functional assays, vaccine/drug testing.	Highly established translational platform; long‐term microbiota and immune–stromal complexity remain challenging.	[113, 114, 115, 116, 117, 118, 119, 120, 121]
Endometrium	Hormone‐responsive glandular epithelial organoids; emerging assembloid, chip, and scaffold‐based variants add stromal/ECM context.	Endometrial physiology, implantation modeling, endometriosis, endometrial cancer, and regenerative studies of thin endometrium/IUA.	Emerging reproductive‐medicine platform; still limited by epithelial bias, incomplete stromal–immune–vascular integration, and hormonal standardization.	[122, 123, 124, 125, 126, 127, 128, 129, 130, 131, 132, 133, 211, 212, 213, 214, 215, 216, 217, 218, 219, 220, 221, 222, 223, 224, 225]

Abbreviations: ADPKD, autosomal dominant polycystic kidney disease; AMD, age‐related macular degeneration; CAKUT, congenital anomalies of the kidney and urinary tract; CFTR, cystic fibrosis transmembrane conductance regulator; CRC, colorectal cancer; CRISPR, clustered regularly interspaced short palindromic repeats; ECM, extracellular matrix; IBD, inflammatory bowel disease; IUA, intrauterine adhesion; LSEC, liver sinusoidal endothelial cell; MASH, metabolic dysfunction‐associated steatohepatitis; RGCs, retinal ganglion cells; RPE, retinal pigment epithelium; SARS‐CoV‐2, severe acute respiratory syndrome coronavirus 2.

## 5 Current Limitations and Unmet Challenges

Although organoid models have substantially improved the physiological relevance of in vitro research, they should not be viewed as complete miniature organs. Their value lies in capturing selected principles of tissue organization, lineage differentiation, disease‐associated phenotypes, and patient‐specific responses rather than reproducing the full anatomical, vascular, immune, neural, and mechanical complexity of native organs. This distinction is important because many current limitations are not simply technical imperfections but reflect the gap between self‐organized tissue fragments and fully integrated organs. The major challenges discussed below are therefore not isolated problems; they are interrelated barriers that determine whether organoids can move from descriptive models toward reproducible and clinically actionable platforms [25, 30].

### 5.1 Lack of Functional Vasculature and Necrosis

The absence of a stable, perfusable vasculature remains among the most fundamental limitations of organoid systems. In vivo, blood vessels provide oxygen, nutrients, endocrine signals, immune cell trafficking, waste removal, and organ‐specific endothelial cues. In contrast, most organoids depend mainly on passive diffusion. As the organoid size or culture duration increases, oxygen and nutrient gradients develop, and the inner regions may become hypoxic, metabolically stressed, or necrotic, which directly limits long‐term maturation and functional readouts [36, 37]. This problem is particularly relevant for brain, cardiac, kidney, liver, retinal, and endometrial organoids, where vascular signals are not merely supportive but actively shape tissue patterning, maturation, and injury responses [74, 85, 90].

Several strategies have been developed to address this limitation. Gao et al. summarized broad vascularization approaches, including endothelial coculture, angiogenic factor stimulation, microfluidic perfusion, transplantation‐induced host vascularization, and bioengineering‐based vessel assembly [5]. In kidney organoids, Homan et al. reported that fluid flow can enhance vascularization and maturation, indicating that physical perfusion cues may be as important as endothelial cell inclusion itself [37]. Kim et al. further demonstrated that a decellularized kidney ECM improves the vascularization and maturation of kidney organoids, suggesting that organ‐specific matrix composition can instruct vascular development [90]. In reproductive models, Ahn et al. established a vascularized endometrium‐on‐a‐chip system, while Shibata et al. and Mol et al. advanced embryo–endometrial interface models that began to incorporate stromal and vascular‐like features [31, 32, 33, 34, 35, 36, 37, 38, 39, 40, 41, 42, 43, 44, 45, 46, 47, 48, 49, 50, 51, 52, 53, 54, 55, 56, 57, 58, 59, 60, 61, 62, 63, 64, 65, 66, 67, 68, 69, 70, 71, 72, 73, 74, 75, 76, 77, 78, 79, 80, 81, 82, 83, 84, 85, 86, 87, 88, 89, 90, 91, 92, 93, 94, 95, 96, 97, 98, 99, 100, 101, 102, 103, 104, 105, 106, 107, 108, 109, 110, 111, 112, 113, 114, 115, 116, 117, 118, 119, 120, 121, 122, 123, 124, 125, 126, 127, 128, 129, 130, 131, 132, 133]. These studies show that vascularization is moving from a generic technical goal toward an organ‐specific design principle.

Nevertheless, current vascularized organoids still rarely contain fully perfusable, stable, hierarchically patterned, and functionally mature vascular networks. Many systems produce endothelial‐like structures but do not reproduce long‐term blood flow, vessel barrier function, pericyte coverage, or organ‐specific endothelial heterogeneity. Research efforts within this field ought to shift focus away from simply verifying vascular formation within organoid models, toward establishing tailored criteria for vascular functionality matched to distinct experimental objectives. Studies examining drug metabolism prioritize sustained perfusion and intact barrier function, investigations of embryo implantation or tissue fibrosis center on signaling interactions between endothelial, stromal and immune cells, while work targeting neural or cardiac tissue maturation places greater weight on trophic factors and mechanical cues originating from vascular compartments. Establishing functional evaluation standards tailored to different research objectives can help avoid relying solely on morphological features to judge vascular structures.

### 5.2 Absence of Immune and Neural Components

Most organoid systems primarily reproduce epithelial or parenchymal compartments and only partially capture immune surveillance, inflammatory remodeling, neural regulation, and stromal crosstalk. This limitation is not trivial because immune and neural components are not accessory elements in many organs. Cross tissue systems display unique multicellular signaling axes: intestinal immune microbial neural crosstalk governs epithelial barrier integrity and inflammatory pathogenesis [113], brain microglia mediate synaptic remodeling and neuroinflammatory processes [71], and endometrial immune cells, stromal fibroblasts, and endothelial cells together with neuroangiogenic signals jointly modulate menstruation, embryo implantation, endometriosis progression, and tissue repair [145, 146, 147, 148, 149, 150, 151, 152, 153, 154].

Different organ systems have addressed this issue with different degrees of progress. Intestinal culture models represent one of the most mature research systems, as coculture setups and organ‐on‐chip devices allow direct characterization of signaling crosstalk between host tissue, resident microbes, and immune populations [115, 116, 117, 118]. Brain organoid studies have made progress in terms of neuronal patterning and assembloids, but the incorporation of microglia, vascular cells, and peripheral immune components in a developmentally appropriate manner remains difficult [70, 71, 72, 73, 74, 75]. In endometrial research, Rawlings et al. developed endometrial assembloids that combine epithelial organoids with stromal cells, and Gnecco et al. used a synthetic matrix‐based coculture system to study epithelial–stromal crosstalk [131, 158]. These models are important because they shift the endometrial field away from epithelial‐only organoids toward more physiologically relevant multicellular systems. However, even these approaches do not yet fully recapitulate the cyclic immune, vascular, stromal, and hormonal microenvironment of the human endometrium.

A practical challenge is that adding more cell types does not automatically improve model quality. Each additional component introduces new variables, including cell source, maturation state, donor matching, culture timing, and relative abundance. Poorly controlled coculture can increase biological noise rather than physiological fidelity. Therefore, immune‐competent or neural‐integrated organoids should be constructed according to the mechanism being studied. For example, embryo implantation research relies on uterine natural killer cells, decidual stromal cells, trophoblast communication, and adaptive vascular structures. Models constructed to investigate endometriosis need macrophages, fibroblasts, endothelial cells, and sensory nerve‐derived factors, while platforms for cancer immunotherapy research require tumor responsive immune cells and physiological immune checkpoint backgrounds [132, 133, 134, 135, 136, 137, 138, 139, 140, 141]. A mature organoid model should not simply contain more cell types but should contain the right cell types in the right state and at the right time.

### 5.3 Heterogeneity, Scalability, and Reproducibility

Organoid heterogeneity remains a persistent obstacle for quantitative biology and clinical translation. Organoids can vary in size, shape, polarity, cellular composition, maturation status, gene expression state, and functional output across donors, tissue sources, matrix batches, passages, and laboratories [6, 9, 26]. Some heterogeneity is biologically meaningful, especially in PDOs that preserve interpatient differences and tumor diversity [21, 24, 130]. However, uncontrolled technical heterogeneity makes it difficult to distinguish true disease‐specific phenotypes from culture‐induced variation.

This issue is particularly evident when mature organoid systems are compared with emerging systems. Intestinal organoids have become relatively standardized because their stem‐cell niche factors, epithelial architecture, and functional assays are well established [15, 113, 114, 115, 116, 117, 118, 119, 120, 121]. In contrast, brain, cardiac, kidney, and endometrial organoids still show substantial variability in maturation, lineage composition, and protocol‐dependent outcomes [70, 71, 72, 73, 74, 75, 86, 87, 88, 89, 90, 91, 92, 93, 94, 95, 96, 97, 98, 99]. Patient‐derived cancer organoid biobanks have shown the translational value of standardized culture and drug testing, but even in oncology, a consensus on organoid‐based drug sensitivity testing remains under development [21, 50, 250]. These comparisons suggest that reproducibility improves when the field defines not only culture recipes but also minimal quality control standards, functional endpoints, and reporting frameworks.

For large‐scale screening, scalability is just as important as biological fidelity. Manual embedding, variable Matrigel droplets, inconsistent organoid size, and subjective imaging analysis limit throughput and reproducibility. Automated culture platforms, high‐content imaging, AI‐assisted phenotyping, and standardized cryopreservation strategies can partly solve these problems [6, 8, 9, 26]. However, technical automation should not replace biological validation. A scalable organoid platform is meaningful only if its readouts remain linked to clinically relevant phenotypes, such as drug response, barrier function, electrophysiology, hormone responsiveness, implantation competence, metabolic activity, or regenerative potential. In this sense, scalability should be treated as a translational requirement rather than simply an engineering convenience.

### 5.4 Ill‐Defined Microenvironment

The microenvironment is both the strength and weakness of organoid culture. Natural extracellular matrices such as Matrigel provide basement‐membrane‐like support and have enabled the establishment of many organoid systems, but they also introduce poorly defined biochemical compositions, variable stiffness, growth factor contamination, and limited clinical compatibility [32, 38]. These issues are particularly problematic when organoids are used for drug screening, mechanistic perturbation, or regenerative applications because matrix‐derived signals may confound pathway interpretation and reduce reproducibility.

Synthetic and tunable matrices offer important alternatives. Gjorevski et al. reported that designer matrices can support intestinal stem cell expansion and organoid formation by controlling matrix mechanics and biochemical ligands [32]. Gnecco et al. extended this concept to the human endometrium, using a fully synthetic ECM to study epithelial–stromal crosstalk in a more defined system [158]. A decellularized ECM represents another strategy, as it preserves tissue‐specific biochemical cues; for example, a kidney decellularized matrix enhances the vascularization and maturation of kidney organoids [90]. These approaches illustrate two complementary directions: synthetic hydrogels improve controllability, whereas decellularized matrices improve organ‐specific biological relevance.

However, neither strategy is perfect. Synthetic matrices may lack the full biochemical complexity of native tissues, whereas decellularized matrices can still vary among donors, organs, and preparation methods [4, 32, 90, 158]. Organ‐on‐chip systems add another layer of control by introducing flow, gradients, mechanical forces, and spatial organization, but they also increase technical complexity and reduce the ease of standardization [25, 30, 106, 133]. Therefore, the next step should not be the universal replacement of Matrigel with a single “better” matrix. Instead, the matrix should be selected according to the biological question. Developmental models may require dynamic stiffness and morphogen gradients; cancer models may require tumor‐specific ECM remodeling; liver and kidney models may require perfusion‐compatible matrices; and endometrial models may require hormone‐responsive stromal‐like mechanisms. A more useful standard for future organoid research is not whether the matrix is natural or synthetic but whether its composition, mechanics, and biological signals are defined well enough to support reproducible interpretation.

## 6 Future Directions

The next stage of organoid research will be defined less by the generation of additional organoid types and more by whether these systems can become physiologically interpretable, technically reproducible, and clinically useful. Over the past decade, the field has moved from proof‐of‐concept self‐organization toward disease modeling, drug screening, regenerative testing, and patient‐specific applications. However, many organoids remain simplified representations of selected tissue compartments rather than integrated organ systems. Future progress should therefore focus on four interconnected goals: improving physiological fidelity, building system‐level models, integrating perturbation and analytical technologies, and establishing standards that allow organoid‐based findings to inform clinical decision‐making [1, 2, 3, 4, 5, 6, 25, 30].

### 6.1 Enhancing Physiological Relevance

A major priority is to improve the structural and functional completeness of organoid models. Current organoids often reproduce epithelial or parenchymal organization but incompletely capture vasculature, immune cells, neural regulation, stromal remodeling, and mechanical forces. This is especially important because these components are not passive background elements; they actively regulate tissue maturation, disease progression, inflammatory responses, and therapeutic sensitivity. Vascularization is among the most urgent challenges. Endothelial coculture, angiogenic factor induction, microfluidic perfusion, transplantation‐assisted vascularization, and 3D bioprinting have been explored as strategies to create more stable vascular networks [5, 25, 30, 36, 37]. Studies in kidney organoids have shown that flow‐enhanced culture can promote vascular maturation, while decellularized extracellular matrices can provide organ‐specific cues for vascular and epithelial development [37, 90]. Similarly, vascularized retinal organoids, vascularized endometrium‐on‐a‐chip systems, and engineered vascular organoid platforms illustrate that vascularization is becoming an organ‐specific design problem rather than a generic culture improvement [51, 85, 133].

With respect to endometrial organoids, improved physiological relevance requires more than the addition of endothelial cells. The endometrium is shaped by cyclic hormone exposure, stromal decidualization, immune cell remodeling, vascular adaptation, and embryo‒epithelium interactions. Recent assembloid, endometrium‐on‐chip, and embryo–endometrial interface models have begun to address these features by integrating epithelial organoids with stromal cells, matrix support, perfusion‐like systems, or embryo/blastoid interaction platforms [68, 131, 132, 133]. These models are particularly valuable because implantation and endometrial repair cannot be understood from the perspective of epithelial behavior alone. In my view, the field should avoid treating “complexity” as an endpoint in itself. A useful organoid model does not need to contain every cell type found in vivo; it needs to contain the components required to answer a specific biological question. For implantation, that may mean epithelial polarity, decidual stromal cells, trophoblast interactions, vascular cues, and uterine immune cells. For fibrosis, stromal fibroblasts, ECM remodeling, inflammatory signals, and mechanical stiffness may be more important. Future models should therefore be designed around mechanism‐driven fidelity rather than maximal cellular complexity.

Matrix engineering will also be central to this transition. Matrigel has been indispensable for establishing many organoid systems, but its undefined composition, variable stiffness, and limited clinical compatibility restrict its mechanistic interpretation and translational use [32, 38]. Designer matrices, tunable hydrogels, organ‐specific decellularized ECMs, and fully synthetic culture systems provide more controllable alternatives [4, 32, 90, 158]. These materials should not simply replace Matrigel as a technical upgrade; rather, they should be used to define how matrix stiffness, ligand density, degradation kinetics, and tissue‐specific ECM composition influence organoid fate. This will be particularly important for studying liver fibrosis, kidney maturation, tumor invasion, and endometrial regeneration, where the ECM is itself part of the disease mechanism.

### 6.2 Multiorgan Integration and Systemic Modeling

Another important direction is the transition from single‐organoid models to multiorgan systems. Human diseases rarely occur in isolation within one tissue. Endocrine disorders, metabolic diseases, cancer metastasis, immune‐mediated diseases, and drug toxicity all involve communication among multiple organs. Multiorganoid chips and linked microphysiological systems offer a way to study these interactions under more controlled conditions than animal models and with more human relevance than standard cell culture [25, 27, 30, 99]. For example, heart–kidney organoid systems can model cardiorenal interactions, whereas gut–liver or liver–pancreas platforms may be useful for studying nutrient metabolism, drug clearance, and systemic toxicity [99, 112].

In reproductive medicine, multiorgan integration has particular potential. The endometrium does not function independently; it is regulated by ovarian steroid hormones, hypothalamic–pituitary signaling, immune status, metabolic state, and embryo‐derived signals. Future models that link ovarian, endometrial, trophoblast, and possibly hypothalamic–pituitary components could provide a more complete platform for studying PCOS, implantation failure, endometriosis‐associated infertility, menopause, and hormone‐dependent endometrial disorders [31, 45, 46, 126, 232]. Such systems would be especially valuable for distinguishing whether a disease phenotype originates from intrinsic endometrial dysfunction, abnormal ovarian endocrine input, or disrupted embryo–endometrial communication. This distinction is difficult to achieve in clinical samples and almost impossible in simple epithelial organoids.

Cancer research will also benefit from multiorgan platforms. Patient‐derived tumor organoids already preserve important features of tumor heterogeneity and drug response [21, 50, 130]. However, tumor progression, metastasis, and therapeutic resistance are strongly influenced by the organ microenvironment. Pairing primary tumor organoids with liver, lung, vascular, immune, or stromal compartments may allow researchers to study metastatic tropism, immune escape, drug metabolism, and organ‐specific toxicity in a single experimental framework. The key challenge is to ensure that these linked systems remain interpretable. Multiorgan models should not become technically impressive but should be biologically opaque. Their value depends on carefully defined inputs, measurable outputs, and validation against patient data.

### 6.3 Technological Convergence: Multiomics, CRISPR, and AI

Future organoid studies will likely combine model engineering with functional perturbation and high‐resolution analysis. Multiomics approaches, especially single‐cell transcriptomics, spatial transcriptomics, epigenomics, proteomics, and metabolomics, can map how different cell states emerge, interact, and respond to disease or treatment [26, 77, 154]. In endometrial research, spatial and single‐cell studies have already shown that epithelial, stromal, immune, endothelial, and metabolic programs change across the menstrual cycle and in disorders such as endometriosis, adenomyosis, thin endometrium, and PCOS [134, 147, 148]. Combining these datasets with organoids will make it possible to test whether disease‐associated signatures are merely correlative or functionally causal.

CRISPR‐based perturbation will be especially useful for this purpose. Genome editing in organoids allows researchers to introduce or correct mutations, perform loss‐of‐function screens, validate disease‐associated genes, and identify therapeutic vulnerabilities [20, 39, 40, 41]. In cancer organoids, this approach can be used to test oncogenic drivers, resistance mechanisms, and synthetic lethal interactions. In endometrial disease, CRISPR‐based tools could help clarify the functional roles of HOX gene methylation, progesterone resistance, PI3K/AKT pathway activation, ferroptosis, epithelial–stromal communication, and fibrosis‐related signaling [155, 156, 157, 158, 168, 169, 170, 171, 172, 173, 174, 175, 176, 177, 178, 179, 180, 181, 182, 183, 184, 185, 186, 187]. The future value of CRISPR‐based organoid systems will depend on moving beyond single‐gene validation toward pathway‐level interpretation, ideally integrated with spatial and functional readouts.

AI will become increasingly useful, but its role should be practical rather than ornamental. AI can assist with automated segmentation, organoid morphology scoring, high‐content imaging, culture optimization, and prediction of drug response [6, 8, 26]. It may also help standardize organoid quality control by extracting features that are difficult to assess manually, such as growth kinetics, lumen formation, branching complexity, polarity, cell death, and differentiation state. However, AI models are only as reliable as the datasets used to train them. For organoid research, imaging, culture metadata, passage number, donor background, matrix type, sequencing data, and clinical annotation must be collected in a standardized way. Otherwise, AI will amplify existing variability rather than solving it. In my view, the most useful AI applications will be those that connect organoid morphology and molecular profiles with functional endpoints, such as drug sensitivity, electrophysiology, barrier function, hormone responsiveness, implantation competence, or regenerative capacity.

### 6.4 Standardization, Biobanks, and Clinical Translation

Clinical translation will require a shift from promising individual studies to reproducible, standardized, and prospectively validated platforms. PDO biobanks have already demonstrated value in research on cancer, cystic fibrosis, liver disease, and gynecological disorders by preserving patient‐specific molecular and functional features [43, 44, 50, 108, 130, 181, 182, 183, 184, 185, 186, 187]. However, biobanks are useful only when linked to high‐quality clinical metadata, consistent culture protocols, reliable quality control, and clinically meaningful endpoints. For oncology, this approach involves comparing organoid drug sensitivity with real patient response in prospective studies. For genetic diseases, the use of organoid‐based functional assays as predictors of therapeutic benefit should be validated. For regenerative medicine, it involves demonstrating engraftment, safety, functional integration, and long‐term efficacy in appropriate preclinical and clinical settings.

Endometrial organoid biobanks deserve particular attention. Endometrial diseases are heterogeneous and hormonally dynamic, and the diseased tissue is often difficult to sample repeatedly in vivo. Biobanks covering the normal cycling endometrium, endometriosis, adenomyosis, PCOS‐related endometrial dysfunction, thin endometrium, intrauterine adhesion, hyperplasia, and endometrial cancer could provide a unified resource for studying disease mechanisms, hormone responsiveness, receptivity, fibrosis, and treatment response [44, 45, 130, 142, 143]. However, researchers in the field should be careful to not equate biobank size with scientific value. A smaller biobank with a well‐defined menstrual cycle phase, hormone exposure, pathology, fertility outcome, treatment history, and omics annotation may be more useful than a large but poorly annotated collection. For reproductive applications, cycle timing and endocrine context should be treated as essential metadata, not optional information.

Standardization must also extend to culture reporting. Future studies should routinely report tissue source, donor characteristics, passage number, matrix composition, medium formulation, hormone treatment, culture duration, organoid size distribution, cell‐type composition, genomic stability, and functional assays. For translational or therapeutic use, good manufacturing practice‐compliant workflows and xeno‐free or chemically defined matrices will be needed [6, 9, 32]. Regulatory frameworks will also need to distinguish among the organoids used as research models, diagnostic tools, drug screening platforms, and cell‐based therapeutic products. These categories have different safety, reproducibility, and validation requirements. Without such distinctions, clinical translation may remain fragmented despite rapid technical progress.

### 6.5 Ethical and Sustainability‐Related Considerations

As organoid models become more sophisticated, ethical and sustainability‐related issues will become increasingly important. Brain organoids and neural assembloids raise questions about neural maturation, sensory input, and the boundaries of ethically acceptable modeling [70, 71, 72, 73, 74, 75]. Embryo–endometrial interface models and postimplantation coculture systems require careful observation because they approach sensitive stages of early human development [31, 46, 132]. Tumor organoid biobanks and patient‐derived disease models also involve genetic data, privacy protection, consent for future use, data sharing, and potential commercialization. These questions should not be treated as obstacles to innovation; rather, they are part of building a trustworthy translational framework.

Cost and accessibility are also practical concerns. Many organoid protocols rely on expensive growth factors, specialized matrices, microfluidic devices, sequencing technologies, and advanced imaging systems. If these platforms remain limited to a small number of well‐funded laboratories, their clinical and global impact will be restricted. More affordable defined media, reusable or scalable culture devices, robust cryopreservation, and simplified quality‐control assays will be necessary for broader adoption [6, 9, 49]. This is particularly relevant for reproductive medicine and rare diseases, where patient populations may be geographically dispersed and sample availability is limited.

In essence, the future of organoid research should not be framed simply as making organoids larger, more complex, or more visually similar to organs. The more important goal is to make them more faithful to the biological question, more reproducible across laboratories, and more predictive of patient‐relevant outcomes. For some applications, this will require vascularized, immune‐competent, multiorgan systems; for others, a simpler but well‐controlled epithelial organoid may be more informative. The field will mature when the model complexity is matched to the experimental purpose. This principle is especially important in gynecology: endometrial organoids, embryo‒endometrial interface models, and reproductive multiorgan systems provide new opportunities to study hormone‐dependent disease, implantation failure, fibrosis, and regenerative repair, but their long‐term value depends on rigorous standardization, clinical annotation, and careful functional validation. If these challenges are addressed, organoids will become not only experimental models but also practical tools for precision medicine, regenerative therapy, and mechanism‐driven clinical decision‐making.

## 7 Conclusions

Organoid technology has become important for modeling human development, disease mechanisms, drug response, and regenerative potential. By integrating stem cell biology, matrix engineering, microfluidics, genome editing, multiomics, and AI, organoids provide a practical bridge between traditional in vitro models and in vivo physiology. For the brain, retina, kidney, heart, lung, liver, intestine, and endometrium, these systems preserve key features of tissue architecture, cellular diversity, lineage organization, and patient‐specific disease phenotypes, supporting more reliable studies of organ development, injury, infection, fibrosis, cancer, toxicity, and therapeutic response.

Despite this progress, compared with complete organs, organoids remain simplified models. Incomplete vascularization, limited immune and neural integration, immature cell states, matrix variability, scalability barriers, and inconsistent standardization continue to limit reproducibility and clinical translation. Future work should focus on building models that are not simply more complex but better matched to specific biological and clinical questions. Defined matrices, vascularized coculture systems, organ‐on‐a‐chip platforms, spatial multiomics, CRISPR‐based perturbation, AI‐assisted phenotyping, and clinically annotated biobanks will be central to this transition. With stronger biological fidelity, technical standardization, and clinical validation, organoids are expected to become practical tools for precision medicine, regenerative therapy, toxicology assessment, and mechanism‐guided drug development (Figure 5).

**FIGURE 5:**
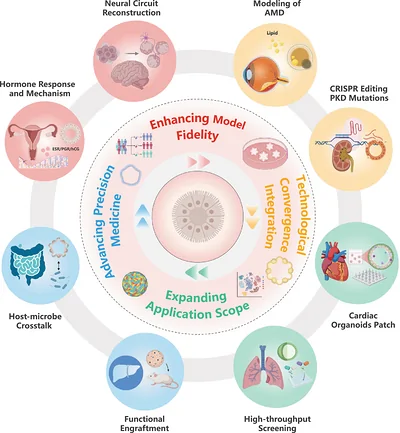
Current applications and future directions of organoids in different organs. Organoid systems enable versatile research across multiple human tissues. Representative current applications include neural circuit reconstruction, age‐related macular degeneration disease modeling, CRISPR‐mediated gene editing for cystic kidney disease mutation study, fabrication of cardiac organoid patches, drug high‐throughput screening, in vivo functional tissue engraftment, host–microbe crosstalk analysis, and mechanistic exploration of tissue hormone responses. Future work will focus on improving model fidelity and expanding application scope. Technological convergence and integration will further advance organoid research toward precision medicine.

## Author Contributions

Conceptualization; data curation; formal analysis; writing – original draft: Yueqi Leng and Yue Wang. Data curation; formal analysis: Canhui Cao and Feng Deng. Data curation; formal analysis: Weisi Lian, Xingtong Chen, and Mingmei Lin. Investigation: Zhonghong Zeng, Dan Mo, and Shangqi Li. Conceptualization: Yilei He and Yang Yu. Conceptualization; funding acquisition; writing – review and editing: Heng Pan, Ping Zhou, and Rong Li. All authors have read and approved the final version of the manuscript.

## Funding

This study was supported by the National Natural Science Foundation of China (82288102, 8257061581, 82271699), and the Beijing Natural Science Foundation (JQ26036, 7252152, 7254445), and the Key Clinical Projects of Peking University Third Hospital (BYSYZD2023028).

## Ethics Statement

The authors have nothing to report.

## Conflicts of Interest

The authors declare no conflicts of interest.

## Data Availability

Data sharing is not applicable to this article as no datasets were generated or analyzed during the current study.
